# The Correlation between Gut Microbiota and Serum Metabolomic in Elderly Patients with Chronic Heart Failure

**DOI:** 10.1155/2021/5587428

**Published:** 2021-10-27

**Authors:** Zhenhua Wang, Zhaoling Cai, Markus W. Ferrari, Yilong Liu, Chengyi Li, Tianzhang Zhang, Guorong Lyu

**Affiliations:** ^1^Department of Cardiology, Second Affiliated Hospital of Fujian Medical University, Quanzhou, Fujian 362000, China; ^2^Department of Internal Medicine 1, Helios-HSK Clinics, Wiesbaden D-65193, Germany; ^3^Department of Ultrasound Medicine, Second Affiliated Hospital of Fujian Medical University, Quanzhou, Fujian 362000, China; ^4^Collaborative Innovation Center for Maternal and Infant Health Service Application Technology of Education Ministry, Quanzhou Medical College, Quanzhou, Fujian 362000, China

## Abstract

**Objective:**

Chronic heart failure (CHF) refers to a state of persistent heart failure that can be stable, deteriorated, or decompensated. The mechanism and pathogenesis of myocardial remodeling remain unknown. Based on 16S rDNA sequencing and metabolomics technology, this study analyzed the gut microbiota and serum metabolome in elderly patients with CHF to provide new insights into the microbiota and metabolic phenotypes of CHF.

**Methods:**

Blood and fecal samples were collected from 25 elderly patients with CHF and 25 healthy subjects. The expression of inflammatory factors in blood was detected by ELISA. 16S rDNA sequencing was used to analyze the changes in microorganisms in the samples. The changes of small molecular metabolites in serum samples were analyzed by LC-MS/MS. Spearman correlation coefficients were used to analyze the correlation between gut microbiota and serum metabolites.

**Results:**

Our results showed that the IL-6, IL-8, and TNF-*α* levels were significantly increased, and the IL-10 level was significantly decreased in the elderly patients with CHF compared with the healthy subjects. The diversity of the gut microbiota was decreased in the elderly patients with CHF. Moreover, *Escherichia Shigella* was negatively correlated with biocytin and RIBOFLAVIN. *Haemophilus* was negatively correlated with alpha-lactose, cellobiose, isomaltose, lactose, melibiose, sucrose, trehalose, and turanose. *Klebsiella* was positively correlated with bilirubin and ethylsalicylate. *Klebsiella* was negatively correlated with citramalate, hexanoylcarnitine, inosine, isovalerylcarnitine, methylmalonate, and riboflavin.

**Conclusion:**

The gut microbiota is simplified by the disease, and serum small-molecule metabolites evidently change in elderly patients with CHF. Serum and fecal biomarkers could be used for elderly patients with CHF screening.

## 1. Introduction

Chronic heart failure (CHF) is a very common multifactor syndrome. Although there are many therapies that are currently used, CHF remains a disease with high morbidity and high mortality. CHF affects not only the heart and blood circulation but also the musculoskeletal, neuroendocrine, metabolic, and immune systems. CHF is associated with altered gut function, including intestinal morphology, permeability, and absorption capacity [1]. Increased gut permeability and bacterial biofilm formation may lead to chronic inflammation and malnutrition. CHF is also considered to be a state of chronic inflammation. The plasma levels of proinflammatory factors, such as tumor necrosis factor- (TNF-) *α*, are related to the severity of disease in CHF patients and can be used to predict survival rates [2, 3]. In fact, it has been found that the wall thickness and permeability of the small intestine and large intestine of CHF patients increase, and the increase in bacterial populations (e.g., *Bacteroides*, *Prevotella*, and *Clostridium*) that adhere to the intestinal mucosa, and the transfer of bacteria or their toxins from the intestine to the blood is directly associated with systemic inflammation [4]. The excessive growth of pathogenic bacteria and *Candida* in the intestine of CHF patients and the increase in intestinal mucosal permeability were found to be related to the severity of clinical disease, venous blood congestion, and inflammation [5]. Chronic systemic inflammation often occurs in CHF patients, and this inflammatory state is closely related to changes in intestinal morphology, function, and bacterial flora.

In addition, recent studies have reported that the gut and the heart are an axis of mutual regulation, and the blood is the bridge between the two interactions [6, 7]. After intestinal barrier function is damaged, bacteria translocate and introduce bacterial products in circulation, which can lead to atherosclerosis and CHF. On the other hand, the damage to heart function in CHF affects the intestinal microcirculation, which leads to intestinal mucosal barrier dysfunction and increased bacterial translocation. The emergence of the heart-gut axis as a new concept once again showed that the gut is closely related to the occurrence and development of cardiovascular diseases [8]. The gut microbiota plays a crucial role in maintaining homeostasis in the host, because its large number of gene products can supplement the physiological processes of the host [9, 10]. Evidence suggests that the gut microbiota has potential clinical significance in the pathophysiology of CHF [11]. Heart failure (HF) patients showed a significant decrease in the diversity of the gut microbiome and downregulation of key gut bacteria. The above studies indicated that changes in the gut microbiome are potential factors involved in the pathogenesis and progression of HF.

The imbalance of gut microbial metabolites leads to intestinal malnutrition, heart dysfunction, and other diseases in CHF patients. There are at least 6,500 low-molecular-weight metabolites in humans, in addition to proteins and genes, that play important roles in biological systems [12]. The latest progress in this field has enabled us to understand the key role of previously unknown metabolites or metabolic pathways in maintaining homeostasis under physiological and stress conditions [13, 14]. The progression of HF promotes the use of glucose rather than fatty acids, and in the late stages of HF, the heart becomes unable to effectively use any of the substrates. In HF patients, due to the decrease in cardiac output or insufficient perfusion, myocardial hypoxia leads to an increase in hypoxia-inducible factor-1*α* (HIF-1*α*), which inhibits pyruvate dehydrogenase (PDH) and promotes cardiac glycolysis without activating glucose oxidation, leading to an increase in the circulating levels of pyruvate and lactic acid and is a prognostic indicator of death [15, 16]. Metabolomics has shown good performance in identifying diagnostic disease markers, which may help to provide new insights into the metabolic processes associated with CHF.

As the global population ages, chronic diseases are placing an increasing burden on public health systems. Studies have shown one to two in every 100 adults in the general population, and more than one in 10 people aged over 70 years are diagnosed with heart failure [17]. At present, there are few studies on the combined use of 16S rDNA sequencing and LC-MS metabolomics to analyze the gut microbiota and serum metabolome in CHF patients, especially the elderly patients. Therefore, our study focused on chronic heart failure in the elderly. In this study, microbiome analysis and metabolomics were used to explore changes in the gut microbiota and serum metabolome during the healthy and CHF to screen effective fecal and serum biomarkers, so as to provide new ideas and insights for elucidating the metabolic process related to CHF.

## 2. Materials and Methods

### 2.1. Study Subjects

Twenty-five CHF patients (H group) and 25 healthy subjects (C group) participated in the experiments. According to current guidelines, the diagnosis of CHF is based on symptoms, clinical signs, and recorded left ventricular dysfunction during exercise. All patients were clinically stable (according to the New York Heart Association (NYHA) functional rating) and had received constant medication for at least 4 weeks prior to evaluation. The study sequentially collected information on medications taken by CHF patients and on the NYHA classification of the disease. All healthy subjects did not take any drugs. The study excluded subjects who had infection, rheumatoid arthritis, renal failure, major valvular heart disease, intestinal disease, cancer, or autoimmune disease. None of the subjects had any known immune system diseases, and none had received immunomodulatory therapy. The clinical data of subjects in different groups are shown in Table 1. The electrocardiogram (ECG) of the 25 healthy and 25 CHF subjects in the healthy and CHF groups was also used as a diagnostic reference of CHF. This study was approved by the ethical review committee of our hospital, and informed consent was obtained from each study participant.

### 2.2. 16S rDNA Sequencing

Fecal samples were collected from 20 healthy subjects and 22 CHF patients by using stool sampling cups. All the fecal samples were stored at -80°C. Microbial genomic DNA was extracted from each fecal sample at 200 mg using the QIAamp ® Fast DNA Stool Mini Kit (Qiagen) according to the manufacturer's protocol. An Agilent 4200 Tapestation Kit (Agilent Technologies) was used to determine the quality of the extracted DNA, and then, the Nextera XT DNA Sample Prep Kit (Illumina) was used to generate sequencing libraries. The bacterial V3/V4 region (F: CCTACGGGNGGCWGCAG and R: GACTACHVGGGTATCTAATCC) was amplified. An Agilent 4200 Tapestation was used to confirm the quality of the libraries, and targeted sequencing of the samples was performed on the HiSeq 2500 platform (Illumina). FastQC (0.11.9) was used for quality control of the original high throughput sequence data. Trim-galore (0.6.5) and CutAdapt (2.8) were used to perform low-quality filtering and joint removal. Cluster analysis was performed with 97% similarity after obtaining effective sequences, and each cluster was called an operational taxonomic unit (OTU). QIIME 2 (2020-2) software and a 16S rDNA sequence database (SILVA-132-99) were used to perform taxonomic annotation and quantitative statistics for the representative sequences of each OTU. A rank abundance curve was used to explain the two aspects of species abundance and species evenness. Qiime 2 software was used to calculate the alpha diversity index (Chao1 index) of each sample and to draw the ranked abundance curve based on ASV and the dilution curve based on alpha diversity. R software was used to draw a boxplot of differences in alpha diversity (R phyloseq package) between groups for the Wilcoxon test (between two groups) method. The MicrobiomeAnalyst platform [18, 19] (https://www.microbiomeanalyst.ca/) was used for bioinformatics analysis.

### 2.3. LC-MS/MS Analysis and Annotation

Blood samples from 25 healthy subjects and 25 CHF patients were used for metabolomic analysis. The blood from all participants was centrifuged at 1150 × g and 4°C for 10 min in a centrifuge tube coated with heparin. The supernatant was then aliquoted (100 *μ*L) into a labeled test tube and stored at -80°C before preparation for metabolomics analysis. Each aliquot (100 *μ*L) was mixed with 300 *μ*L of cold acetonitrile and then vortexed for 30 s before analysis. The mixture was deproteinized by centrifugation at 4°C (21130 × g, 30 mins), and 1 *μ*L of supernatant was injected into a UPLC instrument. After each operation, the needle was cleaned for 5 s. An HPLC system (1290, Agilent Technologies) and a UPLC BEH Amide column (1.7 *μ*m, 2.1 × 100 mm, Waters) combined with a TripleTOF 6600 (Q-TOF, AB Sciex) and a QTOF 6550 were used for LC-MS/MS analysis. The mobile phases consisted of 25 mM NH_4_OAc and 25 mM NH_4_OH (pH = 9.75) (A) and acetonitrile (B) in water. The elution gradient was as follows: 0 min 95% B; 7 min, 65% B; 9 min, 40% B; 9.1 min, 95% B; 12 min, 95% B, delivered at a rate of 0.5 mL/min. The injection volume was 2 *μ*L. In this mode, the acquisition software (Analyst TF 1.7, AB Sciex) continuously evaluates the full-scan survey MS data and triggers the acquisition of MS/MS spectra based on preselected criteria. In each cycle, 12 precursor ions with an intensity greater than 100 were selected and fragmented with collision energy (CE) of 30 V (15 MS/MS events, each product ion had a cumulative time of 50 milliseconds). ESI source conditions were set as follows: ion source gas 1 was 60 psi, ion source gas 2 was 60 psi, curtain gas was 35 psi, source temperature was 650°C, ion spray voltage floating (ISVF) was 5000 V, and positive and negative voltages were 4000 V. The raw data were converted to MZXML format using ProteoWizard and processed using an internal program, which was developed by R software and based on XCMS for peak detection, extraction, alignment, and integration. Then, the internal MS2 database (BiotreeDB) was applied for metabolite annotation. The critical value of the annotation was set to 0.3. QC samples were used to demonstrate the stability of the LC-MS system. MetaboAnalyst [20] (https://www.metaboanalyst.ca/) and Kyoto Encyclopedia of Genes and Genomes [21] (KEGG, https://www.kegg.jp/) were used for bioinformatics analysis.

### 2.4. ELISA

Blood samples were collected from 20 healthy subjects and 22 CHF patients. The level of IL-6 (CSB-E04638h, Cusabio biotech, China), IL-8 (CSB-E04641h, Cusabio biotech, China), IL-10 (CSB-E04593h, Cusabio biotech, China), and TNF-*α* (CSB-E04740h, Cusabio biotech, China) in the blood samples was determined by ELISA kit according to the manufacturer's instructions, and all samples analyses were repeated three times.

### 2.5. Statistical Analysis

Statistical analysis of the data was performed using SPSS 21.0 (IBM, USA) and Graphpad prism 8.0 statistical software. The Gpower software (Cristian-Albrechts University, Kiel, Germany) was used to calculate the experimental validity when selecting samples. All the data are presented as the means ± standard deviations (SDs). Statistical significance between two groups within experiments was determined by unpaired two-tailed Student's *t* tests. A *P* value < 0.05 was considered to be statistically significant. Analysis of similarities (ANOSIM) is a nonparametric test method based on permutation tests and rank-sum tests and is used to test whether the difference between groups is significantly greater than that within groups, so as to judge whether the grouping is meaningful. LDA effect size (LefSe, Galaxy Version 1.0) robustly identifies microbes that are significantly different among biological classes, and additional tests were performed to assess these differences with the LDA value and *P* < 0.05. Spearman correlation coefficients (*P* < 0.05) and heatmaps were used to analyze the correlation among gut microbiota, serum metabolomic, and inflammatory factors by Graph prism (8.0) software.

## 3. Result

### 3.1. The Characteristics of Subjects

**Figure 1:**
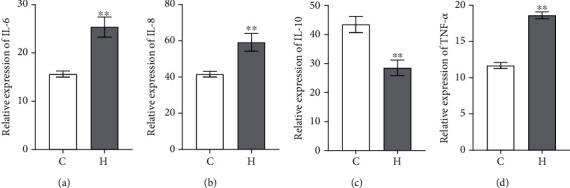
The levels of inflammatory factors in blood were detected. (a) The expression levels of IL-6. (b) The expression levels of IL-8. (c) The expression levels of IL-10. (d) The expression levels of TNF-*α*. ^∗∗^*P* < 0.01 vs. healthy group. C: healthy group; H: CHF group.

Twenty-five healthy subjects and 25 CHF patients participated in this study, and their clinical information was statistically analyzed. The clinical data of all the participants are shown in Table 1. The calculation results of Gpower software showed that the effectiveness of the experiment could reach 80% in the case of 25 cases in each group. The results of clinical data showed that there were no significant differences in age, gender, BMI, or smoking habits between the two groups. The NYHA class disease rating showed that CHF patients had high disease severity. The CRP and NT-proBNP levels of CHF patients were significantly higher than those of healthy subjects. To further analyze the levels of inflammatory factors in CHF patients and healthy subjects, ELISAs were performed, and the results showed that compared with the levels in the healthy group, the levels of IL-6, IL-8, and TNF-*α* in the CHF group were significantly higher, while the expression of IL-10 was significantly lower (Figure 1).

### 3.2. Compositional Alteration of the Gut Microbiota in CHF Patients and Healthy Subjects

**Figure 2:**
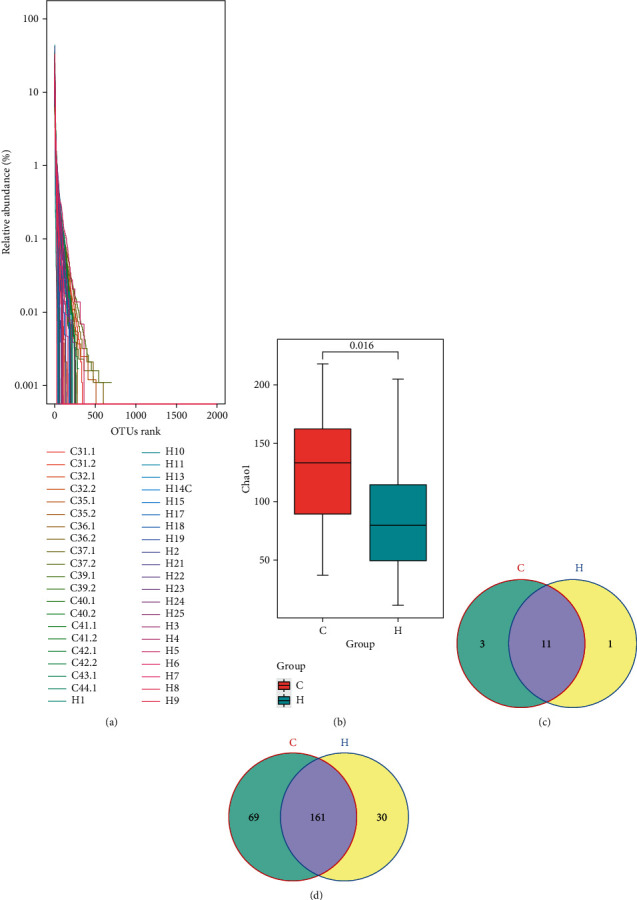
Changes of fecal microbiome. (a) The rank abundance curve. (b) The changes of Chao 1 index. (c) Venn diagram showed microbiota at the phylum level. (d) Venn diagram showed microbiota at genus level. C: healthy group; H: CHF group.

To further analyze whether the structure of the gut microbiota of patients with chronic heart failure has changed, 16S rDNA sequencing was used to detect the fecal microorganisms of CHF patients and healthy subjects. As the OTU rank index increased, the relative abundance gradually decreased, indicating that the species richness and evenness of all the tested samples were high (Figure 2(a)). The rank-sum test of the Chao1 index showed that the fecal microbial diversity of CHF patients was significantly reduced compared with that of the healthy subjects (Figure 2(b)). Taxonomic composition analysis based on OTUs showed that there were 11 common microbial phyla in the feces of healthy subjects and CHF patients (Figure 2(c), Supplementary Figure 1A). There were 3 phylum-specific microorganisms in healthy subjects and 1 phylum-specific microorganism in the feces of CHF patients (Figure 2(c), Supplementary Figure 1A). Analysis of the genera showed that there were 161 common genus-level microorganisms in the feces of the healthy subjects and CHF patients (Figure 2(d), Supplementary Figure 1B). There were 69 genus-level microorganisms in the feces of healthy subjects and 30 genus-level microorganisms in the feces of CHF patients (Figure 2(d), Supplementary Figure 1B). These results indicate that the diversity of the gut microbiota decreased in CHF patients.

### 3.3. Differences of the Gut Microbiota between CHF Patients and Healthy Subjects

**Figure 3:**
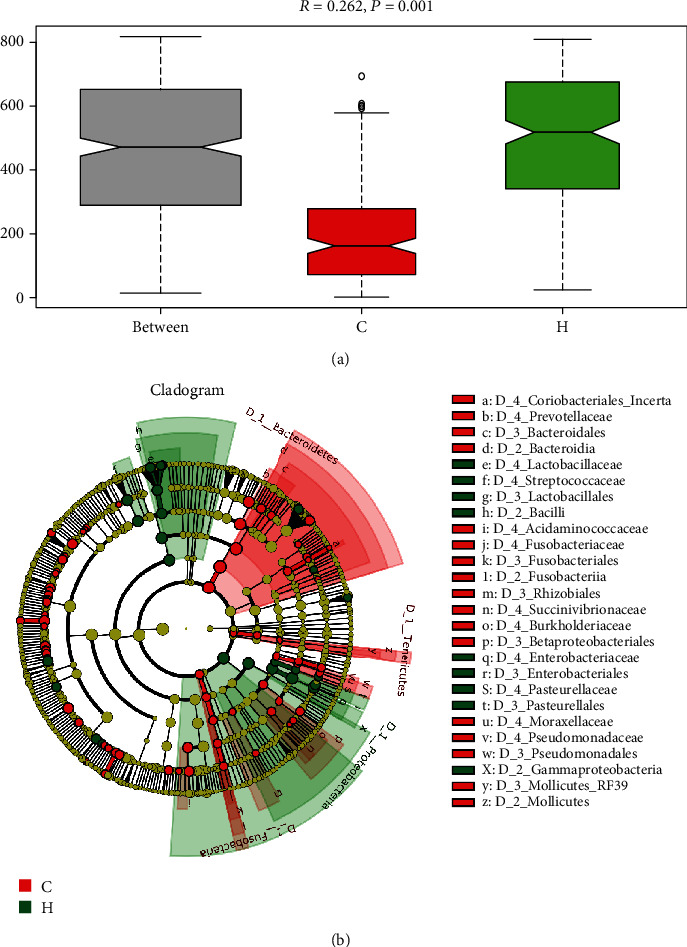
Differential microbial and functional analysis of feces. (a) ANOSIM showed the differentia in groups. (b) Ring-mounted evolutionary branch diagram showed the different microbiota (*P* < 0.05, LDA > 2.28). C: healthy group; H: CHF group.

To investigate taxonomic and functional differences caused by changes in microbial structure, ANOSIM was first used to analyze the differences between groups. ANOSIM showed that the *R* value was 0.262, which is greater than 0 and close to 1, and the *P* value was 0.001, which is less than 0.05, indicating that the difference between groups was greater than that within groups, the difference between groups was obvious, and the grouping was meaningful (Figure 3(a)). To study the differences in the structure of the gut microbiota between groups in depth, LEfSe was used to estimate the impact of the relative abundance of each species on the difference effect and to determine the species with significantly different effects on sample division. The LDA ring-mounted evolutionary branch diagram clearly showed the taxonomic relationship between CHF patients and healthy subjects. The changes at the genus level showed that *Ruminococcus gnavus group* (LDA = 4.42, *P* = 0.0209), *Escherichia Shigella* (LDA = 5.01, *P* = 0.0034), *Ruminococcaceae UCG 005* (LDA = 3.16, *P* = 0.0473), *Ruminococcaceae UCG 002* (LDA = 3.90, *P* = 0.0072), *Lactobacillus* (LDA = 4.50, *P* = 0.0303), *Atopobium* (LDA = 2.48, *P* = 0.0409), *Romboutsia* (LDA = 3.85, *P* = 0.0054), *Streptococcus* (LDA = 4.80, *P* = 0.0122), *Haemophilus* (LDA = 3.10, *P* = 0.0058), and *Klebsiella* (LDA = 4.11, *P* = 0.0018) were significantly enriched in the feces of CHF patients, suggesting that they may be fecal biomarkers for CHF (Figure 3(b)). Functional prediction of gut microbiota showed amino acid metabolism, metabolism of cofactors and vitamins, energy metabolism, replication and repair, and metabolism of terpenoids and polyketides pathways were significantly enriched in the healthy group, and membrane transport and signal transduction pathways were significantly enriched in CHF group at L2 pathways (Supplementary Figure 2A). At L3 pathways, ABC transporters, beta lactam resistance (environmental information processing), and bacterial secretion system pathways were significantly enriched in the healthy group, thiamine metabolism, NOD-like receptor signaling pathway, and beta alanine metabolism pathways were significant enriched in the CHF group (Supplementary Figure 2B). The results showed that the intestinal flora function of the CHF patients had changed.

### 3.4. Serum Metabolomic Profile in CHF Patients and Healthy Subjects

**Figure 4:**
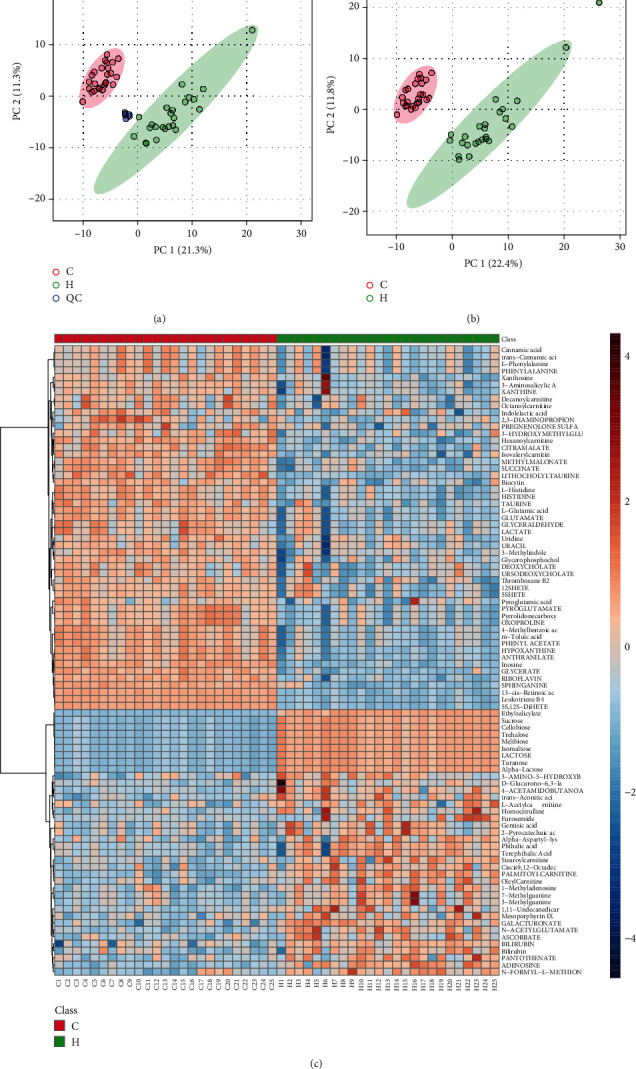
Metabolomic analyses of serum samples. (a) The PCA score plot using the first two components. (b) The PLS-DA scores plot based on fecal metabolic profiles in the healthy and CHF group. (c) Heatmap showed the differential proportion of 90 metabolites in the sample of CHF and healthy groups. The different colors of the circle represent different groups, blue circle: QC sample, green circle: CHF group, red circle: healthy group. C: healthy group; H: CHF group.

We further performed metabolic profiling of serum samples from subjects by using high-throughput liquid chromatography-mass spectrometry (LC/MS). The cluster of QC samples in the PCA score plot demonstrated satisfactory stability and repeatability of the metabolic profiling method (Figure 4(a)). As demonstrated by the PLS-DA score plot, clear separation of control versus CHF samples was observed (Figure 4(b)). Subsequently, heatmap showed a total of 90 potential biomarkers of the CHF group were identified (Figure 4(c)). All of the results indicate that the CHF serum metabolic phenotype was altered compared to the healthy phenotype.

### 3.5. Different Serum Metabolomic of CHF Patients and Healthy Subjects

**Figure 5:**
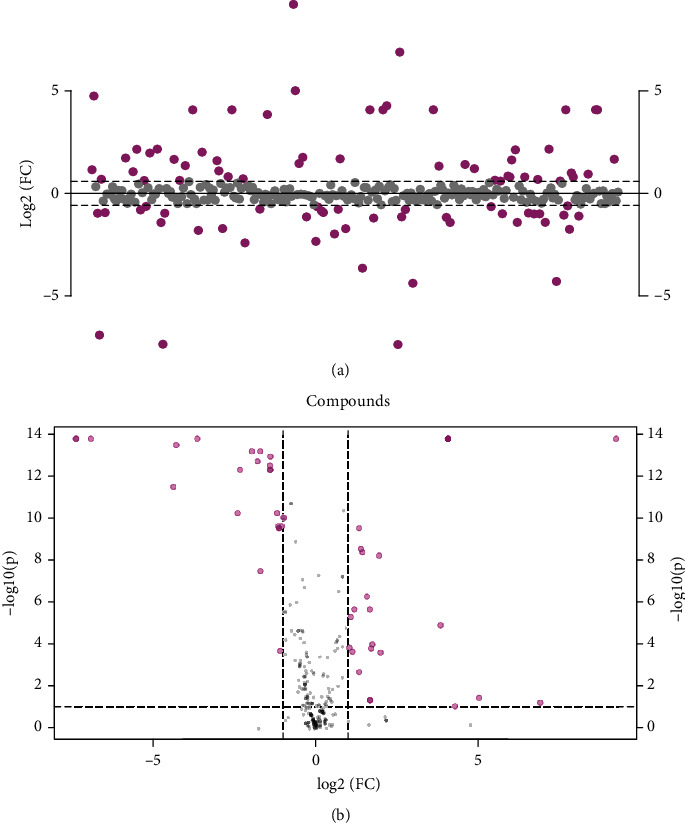
The characteristics and correlation of serum metabolites. (a) The different metabolites associated with CHF were confirmed (log2(FC) > 1.0) (pink). (b) Volcano map showed the different metabolites in CHF and healthy groups.

There are 50 metabolites changed with *P* < 0.05 and log2(FC) > 1.0, including 27 and 23 metabolites that were enriched in the CHF and healthy groups, respectively (Figure 5(a)). The ethylsalicylate, N-formyl-L-methionine, galacturonate, cellobiose, sucrose, turanose, alpha-lactose, lactose, trehalose, isomaltose, melibiose, 3-amino-5-hydroxybenzoate, 1,11-undecanedicarboxylic acid, D-glucurono-6,3-lactone, adenosine, 4-acetamidobutanoate, homocitrulline, N-acetylglutamate, bilirubin, bilirubin, ascorbate, mesoporphyrin IX, 5-methoxysalicylic acid, vanillic acid, 2-pyrocatechuic acid, gentisic acid, and furosemide were significantly enriched in the CHF group (Figure 5(b)). The citramalate, isovalerylcarnitine, pyroglutamate, 13-cis-retinoic acid, 5S,12S-DiHETE, leukotriene B4, inosine, 4-methylbenzoic acid, m-toluic acid, phenyl acetate, anthranilate, hypoxanthine, hexanoylcarnitine, sphinganine, lithocholyltaurine, methylmalonate, succinate, glycerate, glutamate, L-glutamic acid, riboflavin, biocytin, and thromboxane B2 were significantly enriched in the healthy group (Figure 5(b)). All the results indicated that 23 and 27 separately enriched metabolites in the healthy and CHF groups, respectively, may be biomarkers that could contribute to early screening of chronic heart failure patients.

### 3.6. The Correlation among Gut Microbiota, Serum Metabolomic, and Inflammatory Factors

**Figure 6:**
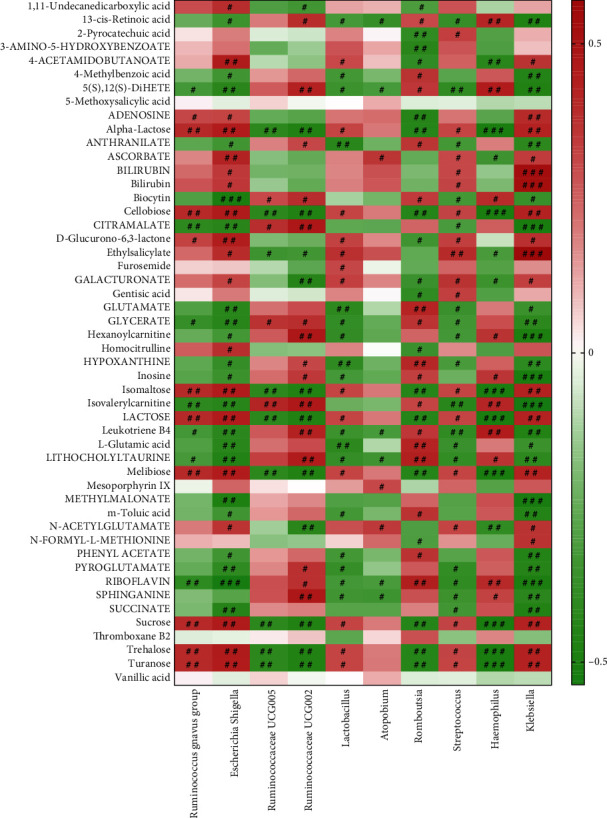
The correlation between gut microbiota and serum metabolomic. Red represents positive correlation, and green represents negative correlation. ^#^*P* < 0.05; ^##^*P* < 0.01; ^###^*P* < 0.001.

**Figure 7:**
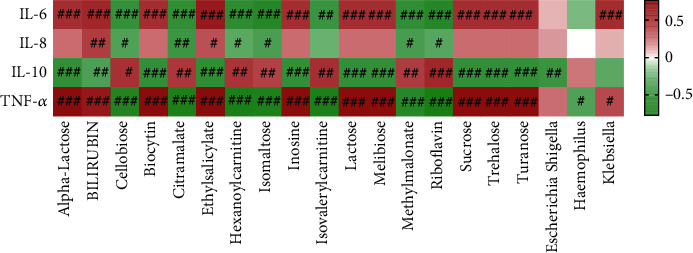
The correlation among gut microbiota, serum metabolites, and inflammatory factors. Red represents positive correlation, and green represents negative correlation. ^#^*P* < 0.05; ^##^*P* < 0.01; ^###^*P* < 0.001.

We analyzed the correlation between gut microbiota and serum metabolomic. We found *Escherichia Shigella* was negatively correlated with biocytin and riboflavin (Figure 6). *Haemophilus* was negatively correlated with alpha-lactose, cellobiose, isomaltose, lactose, melibiose, sucrose, trehalose, and turanose (Figure 6). *Klebsiella* was positively correlated with bilirubin (bilirubin) and ethylsalicylate (Figure 6). *Klebsiella* was negatively correlated with citramalate, hexanoylcarnitine, inosine, isovalerylcarnitine, methylmalonate, and riboflavin (Figure 6). We also analyzed the correlation among inflammatory factors, gut microbiota, and serum metabolites. The results showed that IL-6 was positively correlated with alpha-lactose, bilirubin, cellobiose, ethylsalicylate, isomaltose, lactose, melibiose, sucrose, trehalose, turanose, and *Klebsiella* (Figure 7). IL-6 was negatively correlated with biocytin, citramalate, hexanoylcarnitine, inosine, isovalerylcarnitine, methylmalonate, and riboflavin (Figure 7). IL-8 was positively correlated with bilirubin and ethylsalicylate (Figure 7). IL-8 was negatively correlated with biocytin, citramalate, hexanoylcarnitine, inosine, methylmalonate, and riboflavin (Figure 7). IL-10 was positively correlated with biocytin, citramalate, hexanoylcarnitine, inosine, isovalerylcarnitine, methylmalonate, and riboflavin (Figure 7). IL-10 was negatively correlated with alpha-lactose, bilirubin, cellobiose, ethylsalicylate, isomaltose, lactose, melibiose, sucrose, trehalose, turanose, and *Escherichia Shigella* (Figure 7). TNF-*α* was positively correlated with alpha-lactose, bilirubin, cellobiose, ethylsalicylate, isomaltose, lactose, melibiose, sucrose, trehalose, turanose, and *Klebsiella* (Figure 7). TNF-*α* was negatively correlated with biocytin, citramalate, hexanoylcarnitine, inosine, isovalerylcarnitine, methylmalonate, riboflavin, and *Haemophilus* (Figure 7). All the results showed that gut microbiota, serum metabolomic, and inflammatory factors were correlated in chronic heart failure patients.

## 4. Discussion

In 132 patients with chronic systolic heart failure, plasma measurements of asymmetric/symmetric dimethylarginine (ADMA, SDMA), N-monomethylarginine, and methyllysine have shown that elevated levels of methylated arginine metabolites (demmethyllysine) are associated with left ventricular diastolic dysfunction, and elevated levels of ADMA were associated with later right ventricular systolic dysfunction [22]. Heart failure (HF) and its risk factors promote dysregulation of the intestinal flora, causing damage to the intestinal barrier, and the entry of LPS and other components of the flora into the bloodstream, leading to chronic inflammation, which in turn exacerbates heart failure [7]. It was reported that the structure of the gut microbiota was disordered, and the permeability of the gut was increased in CHF patients [11, 23]. Increased intestinal permeability might cause bacteria and their toxins to enter the blood and cause inflammation [24]. Analysis of inflammation factors showed that the levels of IL-6, IL-8, and TNF-*α* were significantly higher and that of IL-10 was lower in CHF patients than in healthy subjects. As a marker of systemic inflammation, the CRP level was elevated in HF patients, but was within the normal range in healthy controls [5]. The CRP (11.1 ± 2.06 mg/L) and NT-proBNP (6564.5 ± 1187.23 pg/mL) levels of CHF patients were significantly higher than those of healthy subjects. Under normal and healthy physiological conditions, the gut is stable and strictly controlled [25]. Our study showed that the diversity and number of gut microbiota were decreased in the CHF patients, which was consistent with previous studies.

Recently, studies have shown that the gut microbiota can affect the cardiovascular system, and that arrhythmias can aggravate the development of HF [26]. The imbalance of the gut microbiota and intestinal malnutrition was directly related to the origin of various processes of acute or chronic host dysfunction. In addition, studies have reported that the development of CHF is often accompanied by enrichment of pathogens in the gut microbiota [5, 11, 26, 27]. Compared with healthy individuals, CHF patients had more colonizing pathogenic bacteria, including *Campylobacter*, *Salmonella*, *Escherichia Shigella*, *Yersinia enterocolitica*, and *Candida species* [28]. Compared with patients with mild HF, more patients with moderate to severe HF contain *Candida*, *Campylobacter*, and *Shigella species* [28]. Artery atherosclerosis in patients with ischemic stroke and transient ischemic attack case-control study found that patients with stroke and transient ischemic attack more opportunistic pathogens, such as *Enterobacter*, *Megasphaera*, *Oscillibacter*, and *Desulfovibrio*, and fewer commensal or beneficial genera including *Bacteroides*, *Prevotella*, and *Faecalibacterium* [29]. *Eubacterium rectale* and *Dorea longicatena* were less abundant in the HF patients than in that of healthy subjects [30]. Compared to younger HF patients, the *Faecalibacterium* was depleted, while *Lactobacillus* was enriched in the gut of older HF patients [30]. The LDA ring-mounted evolutionary branch diagram clearly showed that *Escherichia Shigella*, *Ruminococcaceae*, *Lactobacillus*, *Atopobium*, *Romboutsia*, *Streptococcus*, *Haemophilus*, and *Klebsiella* were significantly enriched in the feces of CHF patients. Our study found that the more potential pathogenic bacteria and less beneficial bacteria enriched in the gut microbiota of CHF patients. Whether these reduced beneficial bacteria have a potential effect on chronic heart failure remains to be seen. We will further explore the potential role of probiotics or prebiotics in chronic heart failure in animal models, with a view to developing new treatments for chronic heart failure based on the “heart-gut axis” or “gut-heart axis” theory.

Metabolites derived from the intestinal tract and gut microbiota are nutrients that are directly or indirectly digested, absorbed, and utilized by the host [31, 32]. The correlation between gut microbiota and plasma metabolite showed plasma sphingosine 1-phosphate was positively correlated with several CHF-enriched bacteria such as *Veillonella*, *Coprobacillus*, and *Streptococcus*, while plasma-reduced metabolite ricinoleic acid was positively correlated with *Butyricicoccus* enriched in healthy [27]. Our study found *Escherichia Shigella* was negatively correlated with serum biocytin and riboflavin. *Haemophilus* was negatively correlated with serum alpha-lactose, cellobiose, isomaltose, lactose, melibiose, sucrose, trehalose, and turanose. *Klebsiella* was positively correlated with serum bilirubin (bilirubin) and ethylsalicylate, while negatively correlated with serum citramalate, hexanoylcarnitine, inosine, isovalerylcarnitine, methylmalonate, and riboflavin. There were some studies of plasma metabolite in HF disease. Lysophosphatidylcholine 18 : 2, cholesteryl ester 18 : 1, alanine, choline, and fructose were correlated with B-type natriuretic peptide or left ventricular ejection fractions [33]. Orotic acid and N-methylproline were a distinct metabolic signature for differentiated individuals with HF with preserved ejection fraction versus HF with reduced ejection fraction [34]. Norvaline,1-pyrroline-2-carboxylate, lysophosphatidylinositol (16 : 0/0 : 0), phosphatidylglycerol (6 : 0/8 : 0), fatty acid esters of hydroxy fatty acid (24 : 1), and phosphatidylcholine (18 : 0/18 : 3) may have the potential to differentiate patients with dilated cardiomyopathy and ischemic cardiomyopathy, which were common causes of HF [35]. Studies have shown that the gut microbiota in the elderly CHF patients may be related to serum metabolite.

Functional prediction of gut microbiota showed amino acid metabolism, metabolism of cofactors and vitamins, energy metabolism, replication and repair, and metabolism of terpenoids and polyketides pathways were significantly enriched in the healthy group, and membrane transport and signal transduction pathways were significantly enriched in CHF group at L2 pathways. At L3 pathways, ABC transporters, beta lactam resistance (environmental information processing), and bacterial secretion system pathways were significantly enriched in the healthy group, and thiamine metabolism, NOD-like receptor signaling pathway, and beta alanine metabolism pathways were significantly enriched in the CHF group. Studies have shown that skeletal muscle mass and strength are extremely low in 20% of patients with CHF [36]. Supplementation of essential amino acids (EAA) can prevent muscle strength and performance decline and improve quality of life and survival in patients with CHF, which may be a feasible treatment strategy [36]. But we did not analyze the correlation between metabolites and functional pathways due to the limited time and funding issues in this research. In the future, we will perform experiments and analysis to investigate the relationship between microbial function and metabolism.

In conclusion, our study found that the diversity and quantity of gut microbiota in elderly patients with CHF were reduced, the potential pathogenic bacteria were enriched, the beneficial bacteria were depleted, and the changes of serum metabolic map were accompanied. Our study may provide insignificance for the treatment of elderly patients with CHF, but it uncovers the relationship between gut microbiota and serum metabolites in elderly patients with CHF.

## 5. Conclusion

The diversity of the gut microbiota in CHF patients was decreased, and the disease tended to reduce the complexity of microbiota. Imbalance of the gut microbiota and serum metabolites profiles was the basic characteristics in elderly patients with CHF.

## Figures and Tables

**Table 1:** Clinical parameters of subjects.

	CHF (*n* = 25)	Healthy (*n* = 25)	*P* value
Age (years)	65 ± 3.17	65 ± 3.07	0.941
Gender (male/female)	14/11	13/12	
BMI (kg/m^2^)	29.7 ± 1.44	29.1 ± 1.33	0.768
Smoking			0.733
Never	10	12	
Past	11	10	
Current	4	3	
CRP (mg/L)	11.1 ± 2.06	4.22 ± 1.12	0.006
NT-proBNP (pg/mL)	6564.5 ± 1187.23	109.2 ± 45.91	<0.001
NYHA class			
I	2		
II	5		
III	7		
IV	11		

CHF: chronic heart failure; BMI: body mass index; NYHA: New York Heart Association; CRP: cAMP receptor protein; NT-proBNP: N-terminal pro-B type natriuretic peptide. The unpaired *T* test was used between the two groups conforming to the normal distribution. *P* value < 0.05 was considered statistically significant.

## Data Availability

The data that support the findings of this study are available from the corresponding author upon reasonable request.

## Supplementary Materials

Supplementary Materials Supplementary Figure 1: the relative abundance of gut microbiota at phylum and genus level. (a) Relative abundance of microbiota in samples at phylum level. (b) Heatmap showed relative abundance of microbiota in samples at the genus level. C: healthy group; H: CHF group. Supplementary Figure 2: The different pathway at L2 and L3 level in healthy and CHF group. (a) The enriched pathway at L2 level. (b) The enriched pathway at L3 level. C: healthy group; H: CHF group.
