# Supplementary material for: The Correlation between Gut Microbiota and Serum Metabolomic in Elderly Patients with Chronic Heart Failure
Source: Mediators Inflamm. 2021 Oct 27;2021:5587428. doi: 10.1155/2021/5587428 (PMC8566067; doi:10.1155/2021/5587428)
Supplement: Supplementary Materials — Supplementary Figure 1: the relative abundance of gut microbiota at phylum and genus level. (a) Relative abundance of microbiota in samples at phylum level. (b) Heatmap showed relative abundance of microbiota in samples at the genus level. C: healthy group; H: CHF group. Supplementary Figure 2: The different pathway at L2 and L3 level in healthy and CHF group. (a) The enriched pathway at L2 level. (b) The enriched pathway at L3 level. C: healthy group; H: CHF group. [file 5587428.f1.zip › 5587428.f1.pdf]

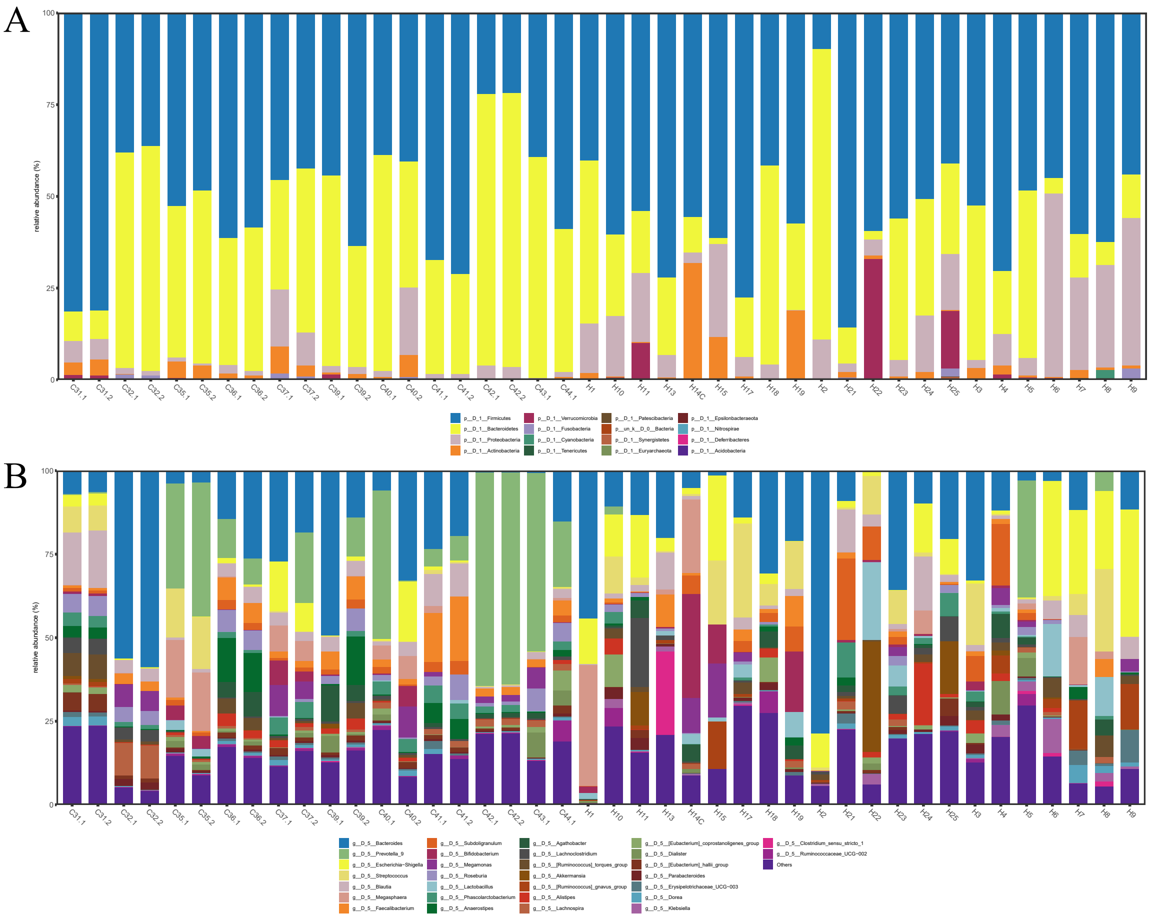

Supplementary Figure 1. The relative abundance of gut microbiota at phylum and genus level. (A). Relative abundance of microbiota in samples at phylum level. (B). Heatmap showed relative abundance of microbiota in samples at the genus level. C: Healthy group, H: CHF group.
